# Body weight at 1.5- and 3-year health checks and body fat at 14 years of age: a population-based retrospective cohort study using dual-energy X-ray absorptiometry

**DOI:** 10.1186/s40101-022-00293-1

**Published:** 2022-05-10

**Authors:** Katsuyasu Kouda, Yuki Fujita, Kumiko Ohara, Harunobu Nakamura, Munkhjargal Dorjravdan, Chikako Nakama, Toshimasa Nishiyama, Masayuki Iki

**Affiliations:** 1 Department of Hygiene and Public Health, Kansai Medical University, 2-5-1 Shin-machi, Hirakata, Osaka, 573-1010 Japan; 2 Center for Medical Education, Kindai University Faculty of Medicine, 377-2 Oono-Higashi, Osaka-Sayama, Osaka, 589-8511 Japan; 3 Department of Health Promotion and Education, Graduate School of Human Development and Environment, Kobe University, 3-11 Tsurukabuto, Nada, Kobe, Hyogo 657-8501 Japan; 4 Department of Public Health, Kindai University Faculty of Medicine, 377-2 Oono-Higashi, Osaka-Sayama, Osaka, 589-8511 Japan

**Keywords:** Adiposity, Children, Densitometry, Epidemiology

## Abstract

**Background:**

In Japan, height and weight measurements, taken for all children at birth and 1.5- and 3-year health checks, are recorded in the Mother and Child Health (MCH) Handbook, as required by the law. The present population-based retrospective cohort study aimed to evaluate the diagnostic performance of height and weight records in the Handbook for predicting excessive adiposity in adolescents.

**Methods:**

The source population consisted of 8th grade students (800 students aged 14 years) registered at two public junior high schools. Of these, we excluded students who were born at a gestational age < 37 weeks or > 42 weeks. The present analyses included 435 participants who provided complete information. Body mass index (BMI) was calculated using height and weight records. Body fat mass at 14 years of age was measured by dual-energy X-ray absorptiometry (DXA). Diagnostic performance of BMI calculated from the MCH Handbook records to discriminate between the presence and absence of excessive adiposity at 14 years of age was evaluated using receiver operating characteristic (ROC) curve analysis. The area under the ROC curve (AUC) was used to quantify the diagnostic accuracy of BMI.

**Results:**

With regard to the prediction of excessive fat at 14 years of age, AUCs and 95% confidence intervals for BMI at 1.5 and 3 years of age were greater than 0.5. Meanwhile, the AUC of BMI at birth was not significantly greater than 0.5.

**Conclusion:**

The present study findings indicate that BMI values calculated using MCH Handbook data have potential ability to distinguish between the presence and absence of excessive fat at 14 years of age.

## Background

Being overweight or obese in adolescence can have adverse consequences such as premature mortality and limited physical mobility in adulthood [1]. Some periods during infancy and early childhood may be critical for the development of obesity in adolescence [2]. One longitudinal study reported that obesity in young adults was more strongly related to weight gain from 1.5 to 5 years of age, whereas birth weight did not predict later obesity [3]. A systematic review concluded that infants who grow rapidly during infancy face an increased risk of later obesity [4]. Unfortunately, most previous studies used body weight to define later obesity as an outcome variable [4], even though obesity is a complex disease involving an abnormal or excessive amount of body fat.

Since body weight includes both muscle and fat, evaluation of fat mass (FM) is more informative than the evaluation of body weight alone. Nevertheless, only a limited number of studies have examined the association between weight as an infant and later FM. One study using correlational analysis reported a positive association between body mass index (BMI) at 3 years of age and whole-body FM as measured by dual-energy X-ray absorptiometry (DXA) at 11 years of age [5]. Another study using correlational analysis reported that rapid infant weight gain was positively associated with DXA-based FM in school-aged students [6]. However, these correlational analyses cannot be used to assess the diagnostic performance of body weight measurements to discriminate between subjects with excessive and normal amounts of FM. Therefore, a categorical analysis, which enables to quantify diagnostic accuracy of weight measurement for distinguishing subjects into clinically relevant subgroups (subjects with excess vs. normal FM), is required [7].

In Japan, free medical examinations are performed for all children at 1.5 and 3 years of age (1.5- and 3-year health checks) in accordance with the Maternal and Child Health Law [8], and all local governments in Japan are obligated to provide these [9]. Height and weight measurements are taken at the 3-year checks, which follow the same format as the 1.5-year checks and are the last of the municipal health checks offered to young children before they begin elementary school (at 6 years of age) [9]. Data from the health checks are recorded in the Mother and Child Health Handbook (MCH Handbook), a tool used by almost all parents in Japan as it serves as a record book shared by parents and health providers to monitor maternal and child health [10]. The MCH Handbook includes records of the pregnancy, delivery, post-partum care, discharge, the newborn’s physiological development, and regular health checkups [11]. The MCH Handbook is distributed by local governments when pregnant women register as such, and nearly 100% of pregnant women use the handbook [12]. Therefore, BMI calculated from height and weight records in the MCH Handbook could serve as useful information that may contribute to the prevention of excessive FM later in life.

No study to date has investigated the clinical performance of BMI calculated from MCH Handbook records, specifically with regard to quantifying its diagnostic accuracy for distinguishing subjects with excessive FM from those with normal FM in adolescence. Receiver operating characteristic (ROC) analysis, a type of the categorical analysis, is useful for quantifying the diagnostic accuracy of classification schemes with one variable and when subjects are classified into two categories with true-positive and false-positive rates [7, 13]. The present study aimed to evaluate the diagnostic performance of height and weight records in the MCH Handbook for predicting DXA-detected excessive FM in adolescence using ROC curve analysis.

## Methods

### Study design and participants

The present study was conducted as a population-based retrospective cohort study using data from the Japan Kids Body-composition Study (JKB Study) [6]. The source population from which our participants were recruited comprised 8th grade students (800 students, mean age of 14 years) registered at Kitakata Municipal Shiokawa Junior High School, Fukushima, in 2010, 2013, and 2016, and at Hamamatsu Municipal Sekishi Junior High School, Shizuoka, in 2013 and 2014. Since there were no other junior high schools in the school district, most students in the given regions attended one of these two schools. Of the source population, 532 students participated in the body composition survey using DXA. Birth weight, which reflects intrauterine growth, increases with increasing gestational age. To investigate an association between birth weight and body fat at age 14 years, potential confounding bias by gestational age needs to be eliminated. Accordingly, we excluded from the analysis participants who were born at a gestational age < 37 weeks or > 42 weeks, on the basis of a previous report [6]. The present analyses included 435 participants who provided complete information.

### Predictive variables

We obtained information regarding body length and weight at birth, body height and weight at 1.5 and 3 years of age, and gestational age at delivery from the MCH Handbook. The information was conveyed to our questioner by parents or guardians of the participants. BMI (kg/m^2^) was calculated as body weight (kg) divided by height squared (m^2^).

### Outcome variables

Body weight, height, and waist circumference at 14 years of age were measured in light clothing and without shoes. Height was measured to the nearest 0.1 cm and weight to the nearest 0.1 kg. BMI cutoffs for overweight and underweight subjects were identified using sex- and age-specific international cutoffs for BMI (overweight: 23.34 kg/m^2^ for girls, 22.62 kg/m^2^ for boys; underweight: 16.88 kg/m^2^ for girls, 16.41 kg/m^2^ for boys) based on adult overweight BMI values of 25 kg/m^2^ and 18.5 kg/m^2^ for girls and boys, respectively [14, 15]. Methods used to measure waist circumference were previously reported in detail [16]. In brief, horizontal waist circumference at the umbilicus level is measured at the end of normal expiration. Whole-body FM was measured with the same DXA instrument (QDR-4500A, Hologic Inc., Bedford, MA, USA) that was brought to the schools in a mobile test room. A single experienced radiological technologist performed all scans and analyses for all participants. Quality control of the DXA scanner was performed on a regular basis using phantoms supplied by the manufacturer. Intra-machine variation calculated from 11 measurements with 2 volunteers was 3.0% (coefficient of variation). Participants removed all metal objects (zippers, belts, snaps, underwire bras, etc.) and their shoes and lay flat on the scanner table during the scan. To identify adiposity, height-normalized index of FM (FM index, FMI; kg/m^2^), which is independent of overall body size, was calculated as whole-body FM divided by height squared (m^2^) [17, 18].

### Statistical analysis

The unpaired *t* test or Mann-Whitney *U* test was used to assess differences between girls and boys. The paired *t* test was used to evaluate differences in BMI from birth to 1.5 years of age and from 1.5 to 3 years of age. Clinical performance of BMI at birth, 1.5, and 3 years of age, specifically with regard to its capacity to discriminate between the presence and absence of excessive FM at 14 years of age, was evaluated using ROC curve analysis [13]. Overall accuracy of BMI at birth, 1.5, and 3 years of age in predicting adiposity at 14 years of age was determined by area under the ROC curve (AUC) and 95% confidence intervals (CI) [13]. In general, a perfect clinical predictor will have an AUC of 1.0, while a completely useless one would have an AUC of 0.5. Two methods (determining the point on the ROC curve closest to the upper left-hand corner (0, 1) and identifying the point corresponding to the maximum Youden index (to maximize the sum of the sensitivity and specificity)) were used to establish the optimal cutoff value of BMI at 1.5 and 3 years of age for predicting excessive FM at 14 years of age [19]. All statistical analyses were performed with SPSS Statistics Desktop for Japan, Version 26 (IBM Japan, Ltd., Tokyo, Japan), with *p* < 0.05 considered statistically significant.

## Results

Participant characteristics are presented in Table 1. BMI values in both girls and boys showed significant increases from birth to 1.5 years of age and significant decreases from 1.5 to 3 years of age. We noted significant sex-dependent differences in BMI at 1.5 and 3 years of age and in FMI at 14 years of age.

**Table 1:** Participant characteristics

	Girls (*N* = 223)	Boys (*N* = 212)	*P*-value^a^
**Birth**			
Gestational age (week)	39.0 (1.1)	39.0 (1.1)	0.71
Length (cm)	48.8 (1.9)	49.4 (1.8)	<0.05
Weight (kg)	3.0 (0.4)	3.1 (0.4)	<0.05
BMI (kg/m^2^)	12.5 (1.1)	12.7 (1.2)	0.12
**1.5 yrs**			
Height (cm)	80.0 (2.8)	81.7 (2.5)	<0.05
Weight (kg)	10.3 (1.0)	11.0 (1.1)	<0.05
BMI (kg/m^2^)	16.0 (1.2)^c^	16.4 (1.3)^c^	<0.05
**3 yrs**			
Height (cm)	95.2 (3.7)	96.8 (3.5)	<0.05
Weight (kg)	14.2 (1.6)	15.0 (1.8)	<0.05
BMI (kg/m^2^)	15.7 (1.1)^d^	15.9 (1.2)^d^	<0.05
**14 yrs**			
Height (cm)	154.7 (5.1)	162.2 (6.7)	<0.05
Weight (kg)	47.2 (7.0)	50.8 (9.7)	<0.05
BMI (kg/m^2^)	19.7 (2.6)	19.2 (2.9)	0.06
Waist circumference (cm)	68.5 (6.5)	68.7 (7.9)	0.73
WtHR	0.44 (0.04)	0.42 (0.04)	<0.05
WtHR > 0.5 (*N*)	22 (10%)	18 (9%)	0.62
BMI > overweight cutoff^b^, (*N*)	18 (8%)	27 (13%)	0.11
BMI < underweight cutoff^b^, (*N*)	21 (9%)	25 (12%)	0.42
Body fat percentage (%)	23.1 (4.9)	14.8 (5.1)	<0.05
FM (kg)	11.2 (3.8)	7.8 (4.1)	<0.05
FMI (kg/m^2^)	4.7 (1.6)	3.0 (1.5)	<0.05

*N*, number; *BMI*, body mass index; *yrs*, years of age; *WtHR*, waist-to-height ratio; *FM*, fat mass; *FMI*, fat mass index

Values represent mean (standard deviation) or *N* (percentage)

^a^*P*-value calculated from the unpaired *t* test or Mann-Whitney *U* test

^b^Determined using age- and sex-specific BMI cutoff points

^cd^*P*-value calculated from the paired *t* test

^c^*P*<0.05 compared to BMI at birth

^d^*P*<0.05 compared to BMI at 1.5 years

**Fig. 1:**
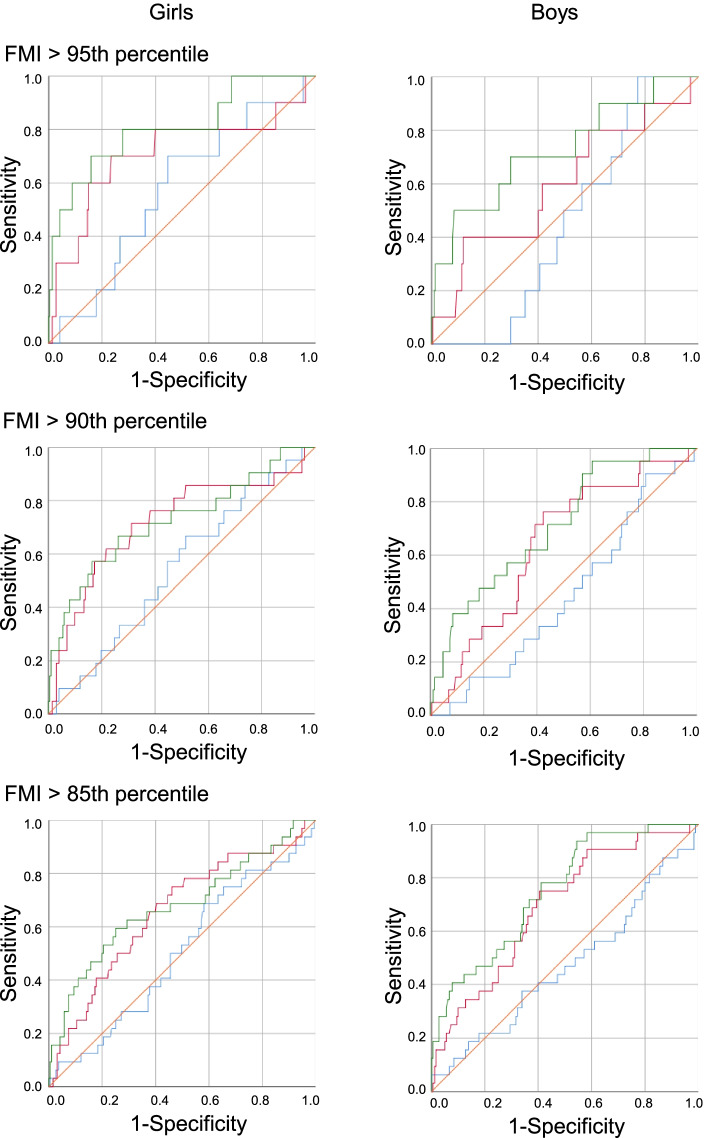
Receiver operating characteristic curves for body mass index at birth (blue line), 1.5 years of age (red line), and 3 years of age (green line) to differentiate between adolescents with and without a high fat mass index (FMI)

Figure 1 shows ROC curves constructed for BMI at birth, 1.5 years of age, and 3 years of age to distinguish between adolescents with and without a high FMI. Table 2 shows the diagnostic accuracy of BMI at birth, 1.5, and 3 years of age for determining whether an individual has excessive FM at 14 years of age. AUCs and 95% CIs generated using data from BMI at 3 years of age and outcomes at 14 years of age (BMI > overweight cutoff, waist-to-height ratio > 0.5, FMI > 95th percentile, FMI > 90th percentile, and FMI > 85th percentile) were greater than 0.5 in both sexes. Similarly, AUCs and 95% CIs for BMI at 1.5 years of age and outcomes at 14 years of age (BMI > overweight cutoff, FMI > 90th percentile, and FMI > 85th percentile) were greater than 0.5 in both sexes. On the other hand, the AUC of BMI at birth was not significantly greater than 0.5.

**Table 2:** Area under the receiver operating characteristics curve

Outcome	Predictor	Girls		Boys	
		AUC 95% CI	*P*-value	AUC 95% CI	*P*-value
BMI at 14 yrs > overweight cutoff^a^	BMI at birth	0.56 (0.43–0.69)	0.38	0.48 (0.36–0.60)	0.73
	BMI at 1.5 yrs	0.78 (0.65–0.90)	<0.05	0.66 (0.55–0.77)	<0.05
	BMI at 3 yrs	0.81 (0.71–0.91)	<0.05	0.75 (0.66–0.85)	<0.05
WtHR at 14 yrs > 0.5	BMI at birth	0.48 (0.35–0.60)	0.75	0.41 (0.30–0.52)	0.20
	BMI at 1.5 yrs	0.68 (0.55–0.81)	<0.05	0.58 (0.45–0.71)	0.26
	BMI at 3 yrs	0.75 (0.63–0.86)	<0.05	0.73 (0.61–0.85)	<0.05
FMI at 14 yrs > 95th percentile	BMI at birth	0.57 (0.40–0.74)	0.45	0.45 (0.34–0.57)	0.61
	BMI at 1.5 yrs	0.71 (0.50–0.91)	<0.05	0.60 (0.40–0.79)	0.31
	BMI at 3 yrs	0.81 (0.65–0.96)	<0.05	0.73 (0.55–0.90)	<0.05
FMI at 14 yrs > 90th percentile	BMI at birth	0.55 (0.42–0.67)	0.49	0.45 (0.33–0.57)	0.45
	BMI at 1.5 yrs	0.72 (0.59–0.85)	<0.05	0.64 (0.53–0.76)	<0.05
	BMI at 3 yrs	0.72 (0.59–0.85)	<0.05	0.71 (0.60–0.82)	<0.05
FMI at 14 yrs > 85th percentile	BMI at birth	0.50 (0.40–0.61)	0.95	0.47 (0.36–0.58)	0.59
	BMI at 1.5 yrs	0.66 (0.55–0.76)	<0.05	0.69 (0.60–0.79)	<0.05
	BMI at 3 yrs	0.68 (0.56–0.79)	<0.05	0.75 (0.67–0.84)	<0.05

*AUC*, area under the receiver operating characteristics curve; *CI*, confidence interval; *BMI*, body mass index; *yrs*, years of age; *WtHR*, waist-to-height ratio; *FMI*, fat mass index

^a^Determined using age- and sex-specific BMI cutoff points

Table 3 presents BMI cutoff points at 1.5 and 3 years of age to identify excessive FM at 14 years of age. True-positive (sensitivity) and true-negative (specificity) rates corresponding to cutoff values are also presented (Table 3). The optimal cutoff value, i.e., the point on the ROC curve closest to the upper left-hand corner (0, 1), was determined as the BMI cutoff point. BMI cutoff points at 3 years of age to screen for FMI at 14 years of age >95th percentile were 16.6 kg/m^2^ for girls and 16.4 kg/m^2^ for boys. True-positive rates were 70% in both girls and boys, and true-negative rates were 84% in girls and 70% in boys.

**Table 3:** BMI cutoff points at 1.5 and 3 years to identify excessive FM at 14 years

	Girls						Boys					
	Cutoff point (kg/m^2^)	Sensitivity	Specificity	Morbidity prevalence rate	Positive predictive value	Negative predictive value	Cutoff point (kg/m^2^)	Sensitivity	Specificity	Morbidity prevalence rate	Positive predictive value	Negative predictive value
**BMI at 1.5 yrs**												
^a^**Closest to upper left-hand corner (0, 1)**												
BMI at 14 yrs > overweight cutoff ^b^	16.7	0.72	0.79	0.08	0.23	0.97	16.4	0.74	0.58	0.13	0.21	0.94
WtHR at 14 yrs > 0.5	16.1	0.77	0.59	0.10	0.17	0.96	16.4	0.61	0.56	0.09	0.11	0.94
FMI at 14 yrs > 95th percentile	16.7	0.70	0.77	0.05	0.14	0.98	16.5	0.60	0.58	0.05	0.07	0.97
FMI at 14 yrs > 90th percentile	16.3	0.71	0.69	0.10	0.20	0.96	16.5	0.71	0.61	0.10	0.17	0.95
FMI at 14 yrs > 85th percentile	16.1	0.69	0.60	0.15	0.23	0.92	16.4	0.75	0.59	0.15	0.25	0.93
^c^**Youden index**												
BMI at 14 yrs > overweight cutoff ^b^	16.1	0.89	0.62	0.08	0.17	0.98	16.4	0.74	0.58	0.13	0.21	0.94
WtHR at 14 yrs > 0.5	16.1	0.77	0.59	0.10	0.17	0.96	15.9	0.83	0.39	0.09	0.11	0.96
FMI at 14 yrs > 95th percentile	16.7	0.70	0.77	0.05	0.14	0.98	17.8	0.40	0.88	0.05	0.15	0.97
FMI at 14 yrs > 90th percentile	16.3	0.71	0.69	0.10	0.20	0.96	16.4	0.76	0.58	0.10	0.17	0.96
FMI at 14 yrs > 85th percentile	15.9	0.75	0.54	0.15	0.22	0.92	16.4	0.75	0.59	0.15	0.25	0.93
**BMI at 3 yrs**												
^a^**Closest to upper left-hand corner (0, 1)**												
BMI at 14 yrs > overweight cutoff ^b^	16.1	0.78	0.74	0.08	0.21	0.97	16.4	0.63	0.73	0.13	0.25	0.93
WtHR at 14 yrs > 0.5	16.1	0.64	0.74	0.10	0.21	0.95	16.4	0.61	0.71	0.09	0.16	0.95
FMI at 14 yrs > 95th percentile	16.6	0.70	0.84	0.05	0.19	0.98	16.4	0.70	0.70	0.05	0.11	0.98
FMI at 14 yrs > 90th percentile	16.1	0.67	0.74	0.10	0.22	0.95	16.4	0.57	0.71	0.10	0.18	0.94
FMI at 14 yrs > 85th percentile	16.1	0.63	0.71	0.15	0.27	0.91	16.0	0.72	0.63	0.15	0.26	0.93
^c^**Youden index**												
BMI at 14 yrs > overweight cutoff ^b^	16.1	0.78	0.74	0.08	0.21	0.97	17.5	0.44	0.94	0.13	0.50	0.92
WtHR at 14 yrs > 0.5	16.7	0.50	0.89	0.10	0.32	0.94	17.5	0.44	0.92	0.09	0.33	0.95
FMI at 14 yrs > 95th percentile	16.6	0.70	0.84	0.05	0.19	0.98	17.5	0.50	0.92	0.05	0.24	0.97
FMI at 14 yrs > 90th percentile	16.5	0.57	0.84	0.10	0.28	0.95	15.5	0.95	0.39	0.10	0.15	0.99
FMI at 14 yrs > 85th percentile	16.1	0.59	0.75	0.15	0.29	0.91	15.6	0.94	0.46	0.15	0.23	0.98

*BMI*, body mass index; *FM*, fat mass; *yrs*, years of age; *WtHR*, waist-to-height ratio; *FMI*, fat mass index

^a^The point on the curve closest to the upper left-hand corner (0, 1)

^b^Determined using age- and sex-specific BMI cutoff points

^c^The point corresponding to the maximum Youden index

## Discussion

In the present population- and DXA-based study, which evaluated the diagnostic performance of BMI at 1.5 and 3 years of age for predicting excessive FM at 14 years of age, both AUCs and 95% CIs calculated from ROC curve analysis were greater than 0.5. The statistical analysis confirmed that these AUCs significantly differed from 0.5, indicating that BMI values calculated using MCH Handbook data have potential ability to distinguish between the presence and absence of excessive FM at 14 years of age. In general, AUC is an effective way to summarize the overall diagnostic accuracy of a diagnostic test [20]. An AUC of 0.5 suggests no discrimination, 0.7–0.8 is considered acceptable, 0.8–0.9 is considered excellent, and above 0.9 is outstanding [20]. The present AUC results, as well as the respective 95% CIs, indicate that BMI at 3 years of age is an acceptable practice to predict excessive FM in the future. On the other hand, the lower limit of 95% CIs at 1.5 years of age was near 0.5, suggesting that the diagnostic performance of BMI at 1.5 years of age is inferior to that of BMI at 3 years of age.

The present study also obtained cutoff values of BMI with the best tradeoff between true-positive (sensitivity) and true-negative (specificity). Unfortunately, neither sensitivity nor specificity of the BMI cutoff points at 1.5 and 3 years of age were remarkably high. To screen for FMI at 14 years of age > 95th percentile, cutoff values for BMI at 3 years of age were 16.6 kg/m^2^ for girls and 16.4 kg/m^2^ for boys, corresponding approximately to the 83rd and 71st percentiles of BMI in girls and boys, respectively. On the other hand, international (International Obesity Task Force) BMI cutoffs for being overweight at 3 years of age, which link a BMI of 25.0 kg/m^2^ (corresponding to the 89th and 91st percentiles of BMI in girls and boys, respectively, at 18 years of age) are 17.6 kg/m^2^ for girls and 17.9 kg/m^2^ for boys [14]. The BMI cutoff values at 3 years of age for predicting excessive FM at 14 years of age were much lower than those used to identify an overweight individual at 3 years of age. Cutoff values for predicting excessive FM at 14 years of age should not be used to discriminate overweight children from normal children at 3 years of age.

To our knowledge, no other study has used ROC curves to assess the utility of BMI in early childhood for predicting adiposity in adolescence, and no study has attempted to identify a cutoff point for BMI in early childhood that might predict adolescent adiposity. Few studies with correlation analysis have examined the association between body weight recorded in MCH Handbook and DXA-based whole-body FM in later childhood [5, 6]. However, the correlational analysis used in those previous studies cannot describe the nature and extent of any misclassifications [7]. A categorical analysis, which enables to quantify diagnostic accuracy of BMI values for distinguishing subjects into clinically relevant categories (subjects with excess vs. normal FM) with true-positive and false-positive rates, is required [7]. For this purpose, the present study used categorical analyses such as ROC curves, and AUCs were used to quantify the diagnostic accuracy of BMI at birth and 1.5 and 3 years of age.

Both the point on the ROC curve closest to the upper left-hand corner (0, 1) and the Youden index are methods commonly used to establish the “optimal” cutoff point with the best tradeoff between sensitivity and specificity. However, the inconsistency in the cutoff points (i.e., the point closest to (0, 1) or the Youden index) was highlighted by our findings. To screen for FMI at 14 years of age > 95th percentile, the cutoff value closest to the upper left-hand corner was 16.4 kg/m^2^ in boys, while the value obtained by the Youden index was 17.5 kg/m^2^. Some inconsistency has been reported in “optimal” cutoff points obtained using these two criteria based on ROC curves [19]. In general, a good cutoff point is one which produces both high sensitivity and high specificity [21]. To minimize the misclassification of true-positives, we recommend that others proceed cautiously when establishing a BMI cutoff point in early childhood for clinical prediction of adolescent adiposity. Further validation studies using larger populations, and those spanning different countries, are required.

This study has several strengths. First, DXA is a safe and simple technique that can be used to determine FM of the whole body in individuals of all ages [22]. The measurement precision is extremely high with DXA [22]. A multicenter study using Hologic QDR 4500 devices reported an inter-instrumental variation of 5.6% (coefficient of variation) for FM [23]. DXA is increasingly being used as a criterion method for body composition assessment and has achieved “reference” status for soft tissue assessment of FM [24]. Second, in the present single-center study, a single radiological technologist performed all scans and scan analyses using the same DXA instrument. Accordingly, our study is free from inter-center variation.

The present study also has some limitations. First, the sample size for the ROC analysis with BMI at 3 years of age to screen for FMI > 95th percentile at 14 years of age was relatively small (9 girls and 10 boys with FMI > 95th percentile). ROC curves created using a smaller sample size are less smooth, which makes it difficult to determine precise cutoff points [25]. However, results for FMI > 95th percentile were consistent with those for FMI > 90th and 85th percentiles, which have a larger number of subjects than FMI > 95th percentile. Second, participants were selected from just a few cities in Japan and thus may be not entirely representative of the entire Japanese population. In other words, we may have introduced some sampling bias. However, according to standard growth charts of Japanese children based on national surveys, mean height/weight measurements of girls and boys at 14 years of age were 155.8 cm/49.3 kg and 162.6 cm/52.5 kg, respectively [26]. Thus, there were no remarkable differences in anthropometric variables between the present population and the general population in Japan. Third, fat accumulation is strongly related to sexual maturity, especially in girls at 14 years of age. However, the growth and developmental status of the participants were not taken into account or considered in the present study.

In the present population- and DXA-based study, ROC curve analysis demonstrated the quantitative accuracy of BMI at 1.5 and 3 years of age for predicting excessive adiposity at 14 years of age, supporting the idea that BMI values calculated using MCH Handbook data have potential ability to distinguish between individuals with and without excessive fat in adolescence. Future studies should investigate which of the childhood BMI cutoff points is most appropriate for predicting adolescent adiposity.

## Data Availability

The dataset generated during the current study is available from the corresponding author upon reasonable request.

## Abbreviations

AUC: Area under the receiver operating characteristic curve
BMI: Body mass index
CI: Confidence interval
DXA: Dual-energy X-ray absorptiometry
FM: Fat mass
FMI: Fat mass index
JKB: Japan Kids Body-composition
MCH: Mother and Child Health
N: Number
ROC: Receiver operating characteristic
WtHR: Waist-to-height ratio
yrs: Years of age
